# Increased Performance Variability as a Marker of Implicit/Explicit Interactions in Knowledge Awareness

**DOI:** 10.3389/fpsyg.2015.01957

**Published:** 2015-12-23

**Authors:** Juliana Yordanova, Roumen Kirov, Vasil Kolev

**Affiliations:** Cognitive Psychophysiology, Institute of Neurobiology, Bulgarian Academy of ScienceSofia, Bulgaria

**Keywords:** serial response time task, implicit learning, explicit knowledge, performance variance, insight

## Abstract

Only some, but not all, individuals who practice tasks with dual structure, overt and covert, are able to comprehend consciously a hidden regularity. The formation of implicit representations of regularity has been proposed to be critical for subsequent awareness. However, explicit knowledge also has been predicted by the activation of executive control systems during task encoding. The present study analyzed performance patterns in participants who could comprehend task regularity and those who could not at delayed recall. Specifically, the role of practice-based knowledge of sequence for individual awareness was focused on. A lateralized variant of the visual serial response time task (SRTT) comprising structured and random blocks was practiced in implicit conditions by 109 participants before and after 10-h retention, with explicit knowledge about covert sequence tested thereafter. Sequence learning was quantified using the normalized difference between response speed in regular and subsequent random blocks. Patterns of performance dynamics were evaluated using response speed, response variability, and error rate. Major results demonstrate that (1) All participants who became aware of the sequence (solvers), gained practice-based sequence knowledge at learning or after retention, (2) Such knowledge also was accumulated during learning by participants who remained fully unaware about covert task structure, (3) Only in explicit solvers, however, was sequence-specific learning accompanied by a prominent increase in performance variability. (4) Specific features and dynamics of performance patterns distinguished different cognitive modes of SRTT processing, each of which supported subsequent knowledge awareness. It is concluded that a behavioral precursor of sequence awareness is the combination of speeded sequence processing and increased performance variability, pointing to an interaction between implicit and explicit processing systems. These results may contribute to refine the evaluation of online and oﬄine learning of tasks with dual structure, and to extend understanding of increased behavioral variability in both normal and pathological conditions.

## Introduction

Continuous information input to the brain contains structured information of environmental regularities. If embedded in a multimodal flow, sometimes this information may not be perceived at encoding and remains out of awareness. The amazing property of the human brain is that structured regularities still can be discovered, either in the course of their repeated presentation (e.g., Frensch et al., 2002) or after a silent period of no exposure to structured information (e.g., Wagner et al., 2004). Conscious understanding of a covert rule leads to a qualitative change in behavior. Awareness of a covert rule may also lead to radically new strategies for problem solving and represents a particularly relevant aspect of creativity (Dietrich and Kanso, 2010).

Experimental conditions exploring gain of awareness typically use tasks with two levels of organization, overt and covert (Haider and Rose, 2007). The overt level is an instructed sensorimotor condition, requiring selective motor responding to pre-defined stimulus conditions. The covert level refers to the presence of a specific regularity in stimulus/response sequences that is unknown to participants. For example, in the typical variant of the serial response time task (SRTT, Nissen and Bullemer, 1987; Robertson, 2007), the overt level takes the form of a visual four-choice reaction task, since there are four response types, each of which is associated with one of four spatial locations of stimulus, and the instruction is to select the correct response as fast as possible. The covert level unknown to participants is the specific sequence of stimulus appearance (e.g., 12 stimuli), which is repeated continuously. In implicit learning conditions, participants may substantially improve performance for the structured sequence without having any expressible knowledge about it (Willingham et al., 1989), or may eventually become aware of it (Nissen and Bullemer, 1987; Ziessler, 1998; Willingham et al., 2000; Destrebecqz and Cleeremans, 2001, 2003). There has been a long standing debate on how this happens and why some but not all individuals have the capability to explicitly discover the regularity (e.g., Reder et al., 2009; Haider et al., 2012; Reber, 2013).

Major models posit that explicit knowledge (ExK) results from accumulated implicit knowledge of regularities. One possible mechanism is that the strength of implicit representations increases in the course of learning. Progressively enhanced implicit sequence representations either remain independent (Willingham et al., 1989) or can be accessed by awareness at a critical level of strength and distinctiveness (Cowan, 1995; Cleeremans and Jimenez, 2002). Alternatively, according to the Unexpected Event Hypothesis (Frensch et al., 2002), the progressive strengthening of implicit sequence representations improves task performance and fluency (Nissen and Bullemer, 1987; Dienes and Perner, 1999; Frensch et al., 2002; Scott and Dienes, 2009). As this occurs unintentionally, altered performance may trigger active conscious exploration of the sources for improvement. Conscious knowledge is thus generated by explicit examination of one’s own behavioral alterations. Explicit search for event sequence also is the principal source of conscious knowledge in explicit learning conditions when subjects are instructed about the presence of regularities or when individual predispositions induce spontaneously cognitive strategies of active search (Robertson, 2007). Whether explicit awareness can emerge passively due to accumulation of implicit representations or it requires active search guided by cognitive control remains an open question.

Recent evidence has shown that individuals who go on to comprehend a hidden regularity (solvers^1^) encode task information differently from individuals who would not comprehend the regularity (non-solvers). Using event-related potentials Lang et al. (2006) have revealed that subsequent solvers, as compared to non-solvers, store the perceived events in memory to a greater extent and pay more attention to the presented stimuli and their sequence. Activation patterns of enhanced cognitive control in solvers have emerged at the very beginning of implicit learning, indicating a trait-dependent difference (Lang et al., 2006; Verleger et al., 2015). Functional MRI and electroencephalographic responses to covert structured information also have revealed a greater activation of executive control regions in future solvers as compared to non-solvers during implicit learning and at test after retention (Yordanova et al., 2009a; Darsaud et al., 2011). Together, these observations indicate that already during learning when task information is encoded, cognitive control mechanisms are more active in those participants who go on to comprehend task structure.

However, predictors of subsequent gain of explicit knowledge have not been clearly identified at the behavioral level. The objective of the present study was to analyze behavioral dynamics during implicit learning of the SRTT and characterize performance patterns in participants who would become solvers at subsequent delayed recall and those who would not. The major hypothesis was that if the ability to bring task knowledge to awareness depends on specific learning strategies, these strategies would be reflected in different performance modes and dynamics. Of special relevance was the question if sequence-specific knowledge acquired implicitly would be critical for subsequent awareness.

In the present study, participants trained a lateralized variant of visuo-motor SRTT implicitly (Verleger et al., 2015), with their explicit knowledge about a hidden sequence tested after a 10-h retention period. To evaluate the progression of learning, material was organized in three successive sub-sessions, in each of which blocks with regular sequences were preceded and followed by random blocks (Cohen et al., 2005). A fourth test sub-session was employed to characterize performance patterns after retention. Performance was evaluated by analyzing the dynamics of several parameters: response speed, performance variance, and error rate (ER) in regular and random blocks. As a marker for sequence-specific learning, the difference between reaction times (RT) in regular and subsequent random blocks was used (Nissen and Bullemer, 1987). RT slowing in random blocks reflects sequence-specific knowledge because of the violation of sequence-based predictions emerging either implicitly (Nissen and Bullemer, 1987) or explicitly (Frensch et al., 2002) during regular block practice.

The present study provides an extended analysis of behavioral data which we reported in a paper on event-related potential predictors and correlates of explicit knowledge and implicit learning in the SRTT (Verleger et al., 2015). Compared to that report, the present paper offers the following. (1) A new classification of participants is introduced, distinguishing between “premature response” and “oﬄine” solvers. (2) The achieved degree of sequence learning is assessed individually in each participant by statistical testing (rather than by median split of the entire group of participants). (3) Variability of RTs is analyzed as a marker of performance. (4) Behavioral data from all blocks in both the pre- and post-retention sessions (after 10 h) are analyzed.

## Materials and Methods

### Participants

One hundred and eleven right-handed and healthy young adults were recruited from a larger study designed to investigate the effects of sleep on hemisphere-specific processing. From these, a total of 109 participants (55 female and 54 male) were used for the present study (mean age 22 years, range 18–31 years). According to the general study design, the retention period after which the gain of explicit knowledge was tested was either of continuous sleep or continuous wake. Accordingly, about half (*n* = 53) of participants performed the task in the morning and the other half (*n* = 56) in the evening (9 a.m. or 9 p.m.), followed 10 h later by a test session in the evening or in the morning, respectively. Also irrelevant to present study was the modulation of side of learning across participants. About half of them trained the task on the left side, and the other half – on the right side. The side of stimulus corresponded to the side of response. Retention and side of learning effects were not analyzed in the present study. All participants were right-handed (evaluated according to the Edinburgh Handedness Inventory, Oldfield, 1971), reported normal or corrected-to-normal vision and no history of chronic somatic, neurologic, or psychiatric disorders. During the experiment no drugs or psychoactive substances were used by the participants. Informed written consent was obtained before the experiment, and participants were paid a flat fee for participating of either 60€ (evening participants who had to stay overnight) or 20€ (morning participants). The study was approved by the Ethic Committee of the University of Lübeck, Germany.

### Serial Response Time Task

**FIGURE 1:**
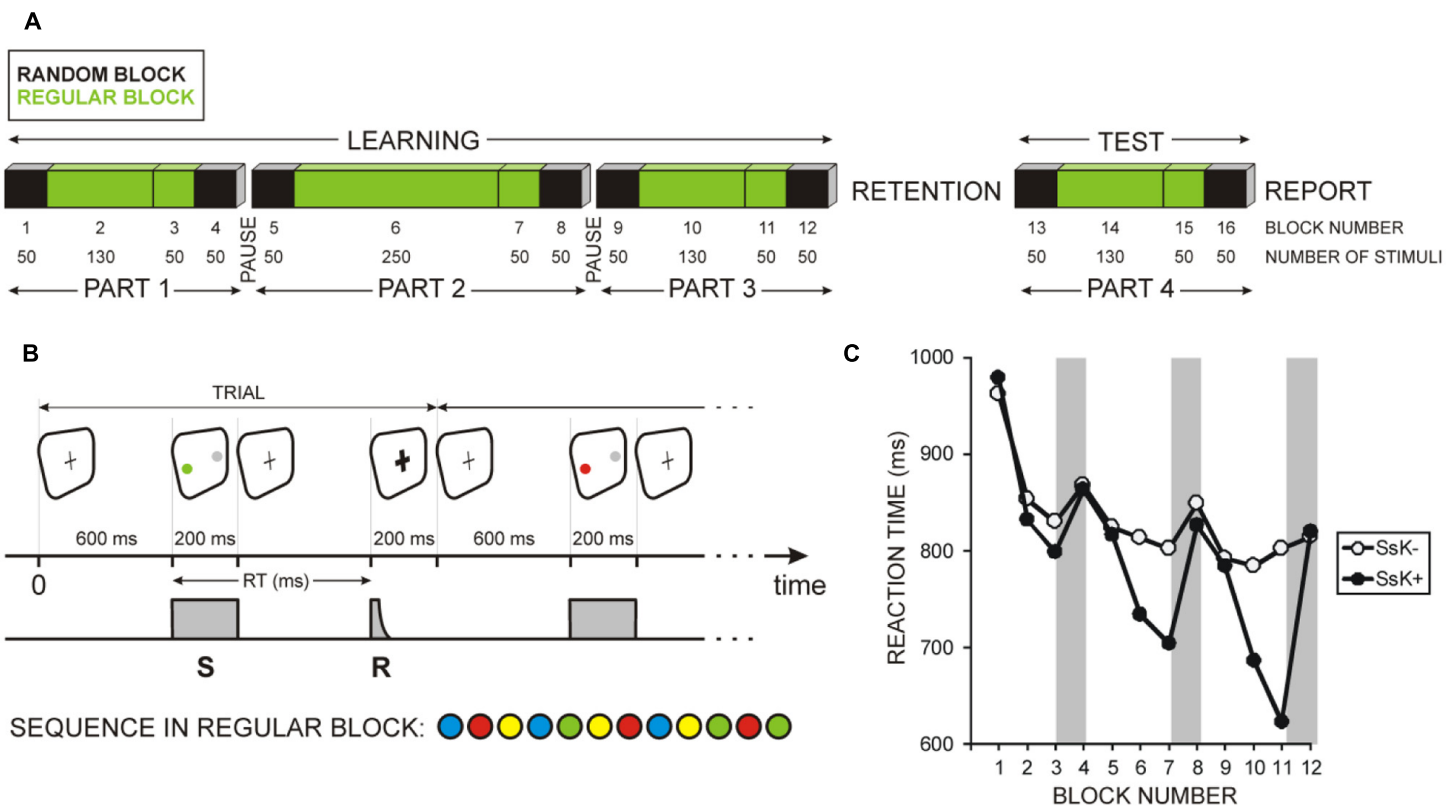
**Graphic illustration of the experiment.** **(A)** Serial response time task (SRTT) was performed in four parts. Unknown to participants, each part was divided in four blocks, here numbered from 1 to 16 (BLOCK NUMBER), with the number of trials in each block indicated (NUMBER OF STIMULI). Blocks indicated in black contained random sequences of stimuli (RANDOM BLOCK); blocks indicated in green contained regularly ordered stimuli (REGULAR BLOCK). Participants practiced SRTT (LEARNING) and performed a TEST session after a 10-h retention period. **(B)** Schematic presentation of one trial of the SRTT. Color stimuli (S) required button-press responses (R) with the respective finger linked to each of the four colors. Sequence in regular blocks was composed of 12 items as indicated. **(C)** Mean reaction times (RTs) for the 12 learning blocks for two groups of participants: (i) who did not gain sequence-specific knowledge during learning (SsK-) and (ii) who acquired sequence-specific knowledge during learning (SsK+). Gray rectangles indicate sequence learning effects – RT difference between the last regular and the last random block in each part of the learning session.

In the study we used a lateralized modification of the SRTT suggested by Nissen and Bullemer (1987) – **Figure 1A**. Stimuli were programmed by means of the Presentation Software version 14.5 (Neurobehavioral Systems, Inc., Albany, CA, USA) and presented on a 17” computer monitor. Participants were instructed to maintain their gaze during the whole experiment to the middle of the monitor. As shown in **Figure 1B**, a fixation cross was permanently visible at screen center (black cross on a white screen). In each trial, two circles of approximately 3 cm^2^ each (diameter of 1°) were presented, one in color and the other in gray, with equal displacement from the screen center of 4.4°. In a given session, the color circles appeared always right or always left, in one of the four colors green, blue, red, and yellow, always counterbalanced by a gray circle at the opposite side. The two circles were presented for 200 ms and the program waited until a button was pressed. If the response was correct, the cross changed after 200 ms for another 200 ms to bold, thus confirming the correctness of execution. Thereafter, the cross returned to its normal shape, and after 400 ms (800 ms after the response) the next color circle appeared. If the response was not correct, the cross did not change to bold and the next color circle did not appear until the correct button was pressed.

To control for eye fixation to the middle point of the monitor, an eye-tracker was used (Eye-Tracker 600 Series, Eyegaze Edge, LC Technologies, Inc., Fairfax, VA, USA). If fixation deviated from screen center by more than 2.6 cm at trial onset (visual angle larger than 1.3°), a large exclamation mark appeared for 2 s in the middle of the screen attracting gaze back to the center. Then the trial was restarted.

Throughout any session, responses were given with the same hand, ipsilateral to the constant side of the color stimuli, by pressing four different buttons. Participants were instructed to press the respective button on a response pad as quickly and accurately as possible. The response pad was designed in such a way that the position of the four buttons corresponded to the position of the fingers of a relaxed freely placed hand: the blue button (B) was exactly below the index finger, the red button (R) below the middle finger, the yellow button (Y) below the ring finger, and the green button (G) below the little finger.

Task structure followed the design used by Cohen et al. (2005). As displayed in **Figure 1A**, from participants’ point of view, the learning session consisted of three parts of 280, 400, and 280 trials, altogether 960 trials, with self-terminated breaks between parts, and the test consisted of one part of 280 trials. One of the four colors appeared in each trial and had to be responded by pressing the appropriate key. Unknown to the participants, each part was a “sandwich” where the outer trials (first 50 and last 50: blocks 1, 4, 5, 8, 9, 12, 13, 16) followed a predetermined quasi-random series (but immediate repetitions of the same color did not occur) whereas the inner trials (180, 300, 180, and 180 in the four parts; blocks 2, 3, 6, 7, 10, 11, 14, 15) repeated a fixed sequence of 12 stimuli (15, 25, 15, and 15 times): B-R-Y-B-G-Y-R-B-Y-G-R-G (**Figures 1A,B**).

### Performance Parameters

For each participant and block (12 learning and 4 test blocks) the following performance parameters were measured: (1) RT to correct responses was calculated as the average of single correct responses. (2) Coefficient of variance (CV) was computed to reflect response variability by dividing standard deviation (SD) of RT by mean RT and multiplying the result by 100. (3) ER reflected performance accuracy and was computed as the percentage of commission error (pressing a wrong key) trials from all trials in a block. (4) For the sake of quantitative group analysis (Nissen and Bullemer, 1987; Robertson, 2009), the rate of RT change in the fourth random block relative to the preceding regular block in each part of the learning and test sessions was computed and used to represent a normalized measure of sequence-specific knowledge (SsK coefficient). (5) Number of correct premature responses in each block was measured. A response was classified as premature if it was faster than 150 ms (Yordanova et al., 2004). This criterion was chosen as being lower than simple reaction task time and indicating that processes which delay RT in four-choice tasks were not executed. Correct premature responses were used to select participants in a separate group.

### Premature Response Group

If a participant had more than 10% premature responses in any of the blocks during learning, he/she was assigned to the premature response group (Prem-R) and was not included in oﬄine explicit knowledge groups detailed below.

### Explicit Knowledge Groups

After the test session, participants filled in a questionnaire to probe their explicit knowledge related to the hidden sequence in regular blocks. They were asked to write on paper any regular sequence they had noted. To quantify the gain of explicit knowledge in the SRTT, participants were scored from 1 to 5 in the following way. In case of no regularity being detected or no feeling of any pattern in the stimulation, the participant was scored with 1. Those who could recall a single sequence of 3–4 items were scored with 2; if they recalled two correct sequences of 3–4 items each, were scored with 3; those recalling a correct sequence of more than eight items were scored with 4, and participants who were able to report the whole sequence of 12 items were scored with 5. With regard to the statistical probability of reporting item sequence correctly, only those who were scored with 3, 4, and 5 were included in the group of explicit solvers (ExK+), whereas those scored with 1 and 2 formed the group of non-solvers (ExK-). It should be noted that ExK groups represent different amounts of explicit sequence knowledge at delayed recall following the test session after retention.

### Sequence Learning Groups

Sequence-specific knowledge (SsK) analyzed here refers to the knowledge about the sequence during SRTT practice in implicit conditions, in contrast to ExK referring to delayed explicit recall of the sequence after retention. To classify participants, gain of SsK was computed at individual level. First, for each participant the Student *t*-test was applied to single-trials in order to determine if RT was significantly longer in the random block than in the preceding regular block (**Figures 1A,C**). In case of significant differences (*p* < 0.05) in the last part 3, the participant was classified as having (SsK+), or not having (SsK-) practice-based sequence knowledge. It is to be noted that SsK+ and SsK- distinction as defined here reflects sequence learning before retention.

### Statistical Analysis

Each statistical parameter (RT, CV, ER, and SsK coefficient) was subjected to repeated measures analyses of variance (ANOVA). To assess performance dynamics in the course of learning, a within-subjects variable Part with 3 levels was included. A second within-subjects variable Regularity was used to contrast regular and random blocks. Performance dynamics at test was assessed using the Regularity variable for four blocks after retention (**Figure 1A**). The between-subjects variables in these analyses were ExK (ExK- vs. ExK+) and SsK (SsK- and SsK+). The objective was to compare performance patterns between participants who would subsequently become aware of the sequence by accounting for their knowledge about the sequence gained while they trained implicitly. Significant group effects and interactions were tested using MANOVA. To characterize learning strategies leading to premature reactions, the dynamics of performance parameters during learning was evaluated for participants with premature responses (Prem-R) in separate analyses with within-subjects variables Part and Regularity, and was compared to that of each other knowledge group using MANOVA (details are presented in the Results). Accordingly, the major SsK × ExK analysis did not include Prem-R participants but only sub-groups of the SsK × ExK combinations as shown in **Table 1**.

**Table 1:** Distribution of participants in knowledge groups.

	ExK-	ExK+	Prem-R	Total number
SsK-	49	14	N/A	63
SsK+	30	9	7	46
Total number	79	23	7	109


*ExK+, gain of explicit knowledge (solvers); ExK-, no explicit knowledge (non-solvers); SsK+, gain of sequence-specific knowledge during learning; SsK-, no sequence specific knowledge during learning; Prem-R, participants with premature responses during the learning session; N/A, not applicable.*

## Results

**Table 1** presents the distribution of participants in different knowledge groups. Seven out of 109 participants were included in the premature response group. In four other participants, only sporadic premature responses were detected for the whole session of 960 trials. All Prem-R participants were aware of the sequence.

### Effects of Sequence Learning on Performance Patterns in Explicit Solvers and non-Solvers

**FIGURE 2:**
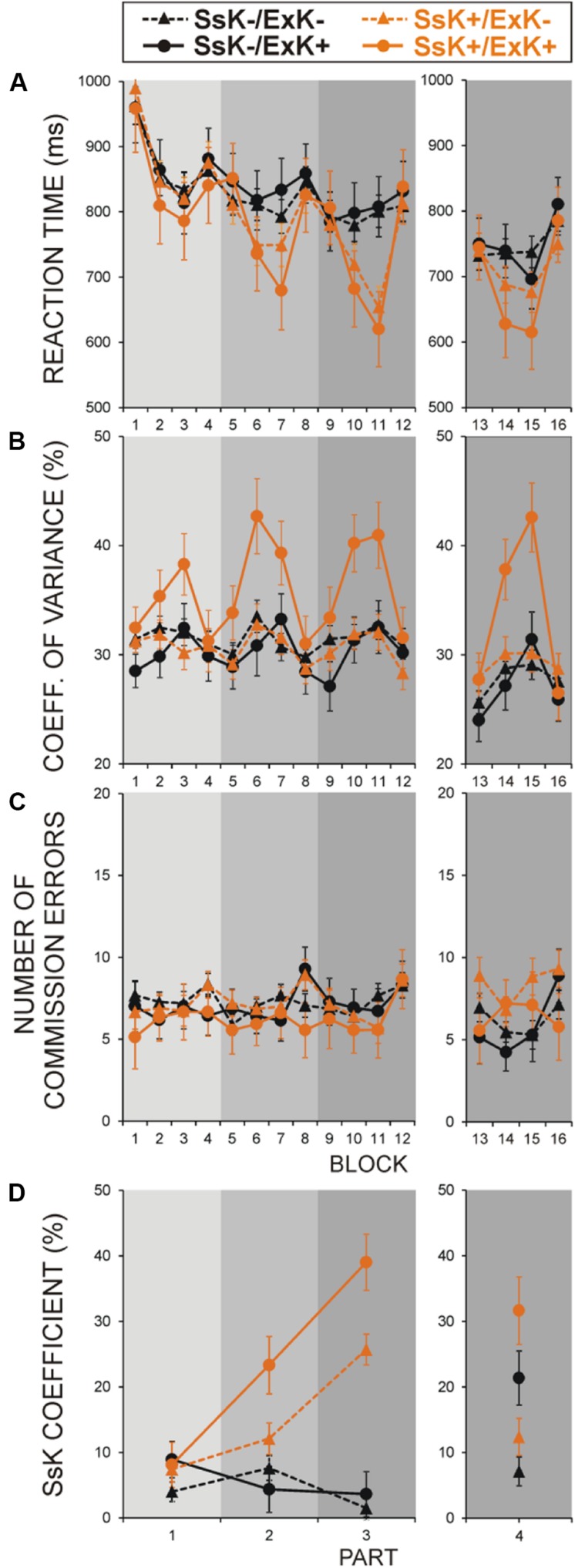
**Effects of sequence-specific knowledge (SsK) gained at learning and oﬄine ExK on performance measures in four groups of participants: (i) non-solvers who did not acquire SsK at learning (SsK-/ExK-), (ii) oﬄine solvers who did not acquire SsK at learning (SsK-/ExK+), (iii) non-solvers who acquired SsK at learning (SsK+/ExK-), and (iv) oﬄine solvers who acquired SsK at learning (SsK+/ExK+).** **(A)** RTs, **(B)** Performance variance, **(C)** Error rate (ER), **(D)** SsK coefficient. Gray rectangles indicate the four parts of experiment as shown in **Figure 1A**. Blocks 1–16 are designated as in **Figure 1A**. Left panels – learning session before retention (blocks 1–12, parts 1–3), right panels – test session after retention (blocks 13–16, part 4).

Dynamics of performance parameters during learning in four sub-groups defined by ExK × SsK interaction is illustrated in the left panels of **Figure 2**.

**Figure 2A** demonstrates that RT in the regular blocks was faster [Regularity, *F*(1/98) = 183.0, *p* < 0.001] and, by definition, was significantly reduced in SsK+ participants [Regularity × SsK, *F*(1/98) = 46.5, *p* < 0.001]. SsK effect was significant in regular blocks of parts 2 and 3 [Part × Regularity × SsK, *F*(2/196) = 16.5, *p* < 0.0001; SsK effect in these blocks, *F*(1/101) = 5.03–15.9, *p* = 0.03–0.001]. Notably, RT did not differ between subsequent solvers and non-solvers, and ExK and SsK factors did not interact [*F*(1/98) = 0.13, *p* > 0.7] in any phase of learning (*p* > 0.3 for any interaction).

In contrast, **Figure 2B** demonstrates that CV depended on whether subsequent solvers (ExK+) have or have not gained sequence knowledge during learning. CV was significantly larger in solvers who acquired sequence knowledge during learning (SsK+/ExK+ sub-group) as compared to solvers who did not (SsK-/ExK+) and non-solvers with or without sequence specific knowledge [SsK × ExK, *F*(1/98) = 4.3, *p* = 0.04]. CV was substantially larger for regular blocks in solvers relative to non-solvers [Regularity × ExK, *F*(1/98) = 13.3, *p* < 0.001] and in the SsK+ than the SsK- group [Regularity × SsK, *F*(1/98) = 7.2, *p* = 0.009], but these effects stemmed from higher CV in only the SsK+/ExK+ group, i.e., explicit solvers who have gained knowledge about the sequence during learning [Regularity × ExK × SsK, *F*(1/98) = 4.3, *p* = 0.04; Regularity × ExK in SsK+, *F*(1/38) = 7.8, *p* = 0.008; in SsK-, *F*(1/62) = 2.8, *p* > 0.1]. No main or interactive effects of ExK and SsK were yielded for ER (**Figure 2C**).

By definition, SsK coefficient was larger in the SsK+ than SsK- group [*F*(1/98) = 52.6, *p* < 0.001] – **Figure 2D**. The difference between ExK+ and ExK- participants [*F*(1/98) = 6.02, *p* = 0.015] resulted from a greater SsK coefficient in the SsK+/ExK+ sub-group [SsK × ExK, *F*(1/98) = 3.8, *p* < 0.05]. Sequence learning in SsK+ participants progressed significantly faster if they, subsequently, went on to discover task regularity [Part × SsK × ExK, *F*(2/196) = 3.4, *p* = 0.036; SsK × ExK in parts 2 and 3, *F*(1/101) > 4.0, *p* < 0.05] – **Figure 2D**.

These results demonstrate that (a) only some of the participants gain practice-based knowledge of the sequence during learning, (b) not all participants who would, subsequently, become aware of the sequence gain practice-based knowledge about that sequence during initial exposure to task, (c) not all participants who learn the sequence through practice can bring this sequence-specific knowledge to awareness, (d) those participants who are able to bring sequence-specific knowledge to awareness are distinguished from all other performers by high performance variability.

### Effects of Sequence Learning on Performance Patterns in Explicit Solvers and non-Solvers After Retention

Dynamics of performance parameters at test after retention (four blocks) in four sub-groups defined by ExK × SsK interaction is illustrated in the right panels of **Figure 2**. **Figure 2A** demonstrates that after retention, RT was faster in the regular than random blocks [Regularity, *F*(1/98) = 91.4, *p* < 0.001]. This effect was significantly more pronounced in ExK+ than ExK- participants (Regularity × ExK, *F*(1/98) = 15.9, *p* < 0.001] and in those who had gained sequence-specific knowledge during learning [Regularity × SsK, *F*(1/98) = 16.0, *p* < 0.001]. For those who had not (SsK-), a significant RT reduction in regular blocks after retention was yielded only in explicit solvers [Regularity × ExK in SsK-, *F*(1/61) = 5.6, *p* = 0.02; Regularity effect in the SsK-/ExK+ group, *F*(1/13) = 8.1, *p* = 0.01; regular block 3 vs. first random block in SsK-/ExK+, *p* < 0.01]. The four sub-groups did not differ in the first random block [SsK × ExK, *F*(1/101) = 0.052, *p* > 0.8], or in the last random block after retention [*F*(1/101) = 0.004, *p* > 0.9].

**Figure 2B** (right) demonstrates that after retention, CV was larger in regular than random blocks [Regularity, *F*(1/98) = 56.1, *p* < 0.001], in the ExK+ than ExK- group [Regularity × ExK, *F*(1/98) = 21.4, *p* < 0.001] and in the SsK+ than SsK- group [Regularity × SsK, *F*(1/98) = 8.2, *p* = 0.005]. As during learning, the latter effects were due mainly to the SsK+/ExK+ participants [Regularity × SsK × ExK, *F*(1/98) = 10.4, *p* = 0.002]. Among other sub-groups CV increased in the third regular block as compared to other blocks only in the SsK-/ExK+ participants [*F*(3/39) = 5.5, *p* < 0.01].

After retention, commission ER was higher in the regular blocks in participants who have acquired sequence knowledge at learning [Regularity × SsK, *F*(1/98) = 6.5, *p* = 0.01], whereas it was decreased in the SsK-/ExK+ group [Regularity × SsK × ExK, *F*(1/98) = 4.3, *p* = 0.04] – **Figure 2C**, right.

As indicated in the right panel of **Figure 2D**, sequence knowledge reflected by SsK coefficient after retention was greater in participants who have acquired this knowledge already during learning [SsK, *F*(1/101) = 4.27, *p* = 0.04], as well as in subsequent solvers [ExK, *F*(1/101) = 19.8, *p* < 0.001]. Consistent with RT finding, a prominent enhancement in sequence knowledge after retention is observed in the SsK-/ExK+ group.

These results show that (a) explicit solvers who had not learned the sequence by practice before retention (SsK-/ExK+) manifest sequence knowledge after retention, (b) increased performance variability in regular blocks after retention remains a distinguishing characteristics of explicit solvers who had accumulated sequence knowledge during learning before retention (SsK+/ExK+).

### Performance Patterns of Premature-Response Participants

Performance parameters in the Prem-R group were assessed using ANOVA with within-subjects variables Part (three levels) and Regularity (regular vs. random blocks). Additionally, the Prem-R group was contrasted with other ExK × SsK knowledge sub-groups (SsK-/ExK-, SsK-/ExK+, SsK+/ExK-, and SsK+/ExK+) using MANOVA.

**FIGURE 3:**
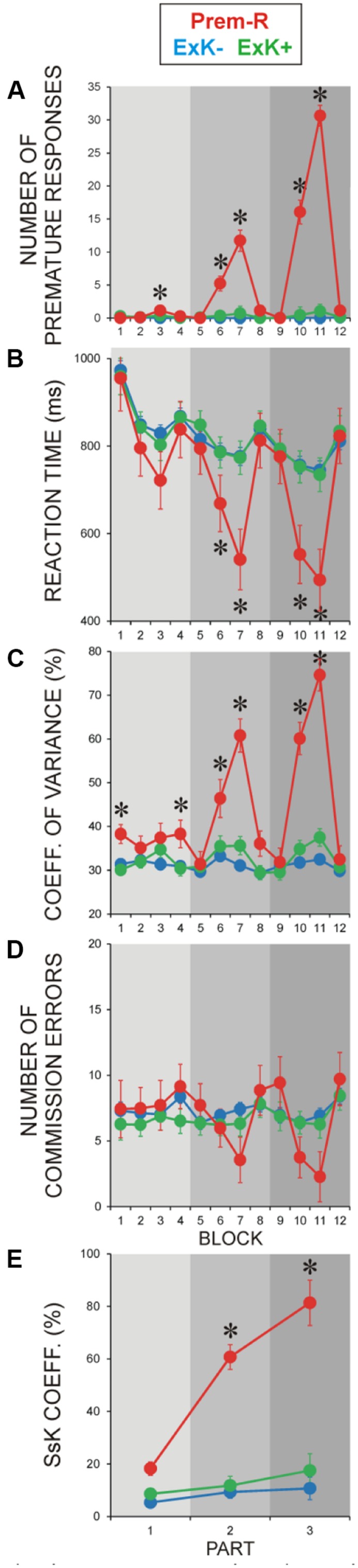
**Performance parameters during pre-retention learning in participants with premature responses (Prem-R).** Oﬄine explicit solvers (ExK+) and non-solvers (ExK-) are only illustrated to provide reference to Prem-R values and dynamics. **(A)** Number of premature responses during the learning session. **(B)** RTs, **(C)** Performance variance, **(D)** ER, **(E)** Sequence-specific knowledge (SsK) coefficient. Gray rectangles indicate three parts of experiment as shown in **Figure 1A**. Blocks 1–12 and parts 1–3 are designated as in **Figures 1A** and **2**. Asterisks indicate blocks, in which the Prem-R group differed significantly (*p* = 0.05–0.001) from each of the four SsK × ExK sub-groups. Between-group differences were tested by MANOVA.

Number of premature responses is shown in **Figure 3A** to verify the selection of the premature response group and to demonstrate that premature responses (a) were generated in the regular blocks [Regularity, *F*(1/6) = 7.5, *p* < 0.05], and (b) appeared in the Prem-R groups already in the first part of learning and increased with learning progression [Part, *F*(2/12) = 10.5, *p* = 0.005; Regularity × Part, *F*(2/12) = 10.3, *p* = 0.003]. Accordingly, in the regular blocks of each part, the difference between Prem-R and other sub-groups was significant [*F*(4/104) = 3.4–86.4, *p* = 0.009–0.001] as indicated in **Figure 3A**.

As demonstrated in **Figures 3B–E**, following the dynamics of premature responses, RT, CV, ER, and SsK coefficient in Prem-R participants manifested significant variations in regular blocks. Accordingly, RT was substantially faster, CV was larger, and ER was smaller in the regular than random blocks [Regularity, *F*(1/6) = 7.4–36.3, *p* = 0.03–0.001], with these effects increasing with practice progression [Part, *F*(2/12) = 7.9–36.1, *p* = 0.008–0.001; Regularity × Part, (*F*(2/12) = 5.1–13.9, *p* = 0.05–0.002], which also was reflected by the SsK coefficient [Part, *F*(2/12) = 14.1, *p* = 0.001].

Consistent with these observations, significant differences between Prem-R and other four knowledge sub-groups groups were yielded for the regular blocks of learning parts 2 and 3 [*F*(4/104) = 3.5–34.9, *p* = 0.04–0.001], with effects not reaching significance for ER (**Figure 3**). Statistical differences between Prem-R and each other group indicated in **Figure 3** were corrected using Bonferroni procedure (*p* = 0.03–0.001). As an exception of these effects in regular blocks modulated by premature responses, response variability in the Prem-R group was significantly increased relative to other groups already in the first part of learning in random blocks when no premature responses were generated by any group [*F*(4/104) > 3.5, *p* < 0.01 for blocks 1 and 4]. Especially for the first random block of learning, CV was significantly larger in Prem-R as compared to each other group (Bonferroni corrected *p* = 0.04–0.004).

These results show that in Prem-R participants, sequence processing during learning is improved in terms of speed (fast/premature responses) and accuracy (decreased ER). Notably, these participants manifest increased performance variability at initial exposure to task.

## Discussion

Only some, but not all, individuals who train on tasks with dual structure, overt and covert, are able to consciously experience the covert task information. This individual ability has been associated with active cognitive control and enhanced consciousness during learning (Lang et al., 2006; Darsaud et al., 2011; Verleger et al., 2015). On the other hand, initial accumulation of implicit sequence-specific knowledge is proposed to be critical for making the covert task information accessible by awareness (Nissen and Bullemer, 1987; Haider and Frensch, 2005, 2009; Haider and Rose, 2007). To assess if such implicit-explicit interactions shape specific learning strategies, the present study analyzed performance patterns in participants who could comprehend a hidden task structure and those who could not at delayed recall. Specifically, we focused on the predictive role of practice-based knowledge of regularity for subsequent awareness. Dynamic changes of a set of performance parameters were analyzed to characterize inter-individual differences in learning strategies at the behavioral level.

According to major results, (1) all participants who became aware of the sequence (solvers), manifested practice-based sequence knowledge, (2) notably, a similar sequence specific knowledge also was accumulated by participants who remained fully unware about the covert task structure, (3) only in explicit solvers, however, was sequence-specific learning accompanied by a prominent increase in performance variability, (4) specific features and dynamics of performance patterns distinguished different cognitive modes of SRTT learning, each of which supported subsequent knowledge awareness, but they all were uniquely characterized by increased performance variability.

### The Role of Practice-Based Sequence Learning for Explicit Knowledge Generation

Practice-based knowledge of the sequence was evinced in all solvers. It was gained either during learning (SsK+/ExK+), or after retention (SsK-/Exk+). This observation confirms the notion that the formation of implicit associative sequence representations is crucial for subsequent awareness of regularity, emphasizing the role of the implicit-to-explicit transition (Frensch et al., 2002; Haider and Rose, 2007; Sun et al., 2007).

However, in a sub-group of participants (SsK+/ExK-), practice-based sequence learning also was found, but these participants remained fully unaware of SRTT regularity. This observation contributes to the debate on whether implicit and explicit systems can operate separately (Schacter, 1992; Reber and Squire, 1994; Seger, 1994; Destrebecqz and Cleeremans, 2001; Forkstam and Petersson, 2005; Abrahamse et al., 2010; Haider et al., 2011; Fu et al., 2013; Reber, 2013) by confirming the existence of stabilized implicit representations of the sequence independent from explicit representations of the sequence (Willingham et al., 1989). Also, this result shows that the mere formation of implicit representations during practice may not be a precursor of conscious comprehension. It may be argued that implicit representations had not reached a threshold or had strength insufficient to tag explicit processing (Cleeremans and Jimenez, 2002). However, the amount of sequence knowledge in the SsK+/ExK- sub-group did not differ from that of SsK+/ExK+ participants in the end of learning, nor did it differ from that of SsK-/ExK+ participants after retention. Hence, below-threshold strength of representations might not be the source of the inability of the SsK+/ExK- subjects to access explicitly accumulated implicit knowledge. It still may be that implicit sequence knowledge in this sub-group can further be strengthened with additional practice, so that access to the explicit system would be reached at a later stage. However, the reduction in sequence knowledge after retention in this sub-group (as seen in **Figure 2**) does not support this possible development. Nor is it certain that oﬄine learning during retention can strengthen additionally implicit sequence representations (Song et al., 2007; Nemeth et al., 2010; Al-Sharman and Siengsukon, 2014). Thus, a substantial gain of implicit sequence specific knowledge may not be on its own a reliable precursor of subsequent awareness. Rather, as will be discussed below, the operationalization of implicit practice-based representations within explicit system functioning appears to be critical.

Comparing sequence learning in explicit solvers and non-solvers shows that in implicit learning conditions, practice-based sequence knowledge can be acquired in different ways. Sequence learning in non-solvers (SsK+/ExK-) emerged on the background of speeded and highly stable and fluent overt performance pointing to proceduralization and automatization of behavior. In contrast, sequence learning in solvers (SsK+/ExK+) was marked by similarly fast but highly variable responses in regular blocks, which also was observed in the SsK-/ExK+ solvers after retention. In explicit solvers with premature responses, performance variability was markedly enhanced by highly speeded or premature reactions in regular blocks (**Figure 3**), Although different mechanisms may be responsible for raised variance in separate sub-groups of solvers (Frensch et al., 2002), the present results are relevant in showing that speeded sequence processing alone may not differentiate the overt level of processing in subsequent solvers and non-solvers. Rather, increased response variability emerging in parallel with advanced sequence learning provides a distinction. From this perspective, the unexpected experience of variation (e.g., disruption, slowing, or conflict) within a fluent proceduralization may generate a neurophysiological signal of mismatch which may act as an efficient online or oﬄine trigger of the explicit processing system.

### Sources of Delayed Explicit Knowledge Generation

Present results reveal that the combination of practice-based sequence learning and increased performance variability provides a marker for conscious comprehension of the sequence. Variants of this combination helped to identify three different types of processing strategies during learning, all of which were associated with the ability to bring hidden task regularity to awareness. These different processing strategies were expressed in the groups of Prem-R solvers, and solvers who did (SsK+/ExK+) or did not (SsK-/ExK+) acquire sequence knowledge at pre-retention task practice.

#### Online Solvers

Haider and Frensch (2005, 2009) and Haider and Rose (2007) propose that in tasks with dual structure (overt and covert), conscious comprehension of the covert rule leads to an abrupt qualitative alteration of behavior, which is marked by a sudden substantial decrease in performance speed (RT drop). Frensch et al. (2002) observed in the number reduction task (NRT) that RT variance of participants who gained insight to the covert rule, increased shortly before they became aware of the rule. This phenomenon was linked to the Unexpected Event Hypothesis and to the switch to an intentional active search for regularities in the stimulus/response material, i.e., to the functioning of a new task-(meta-)representation engaging cognitive control and explicit processing (Haider et al., 2011).

In the currently employed SRTT version, a sub-group of participants (6.4%) produced extremely fast responses faster than 150 ms analogous to “RT drop.” Since this response speed corresponds to simple RT (Yordanova et al., 2004), it is indicative for the fact that processes delaying RT in four-choice tasks (stimulus identification, stimulus-response integration, and response selection), are not executed. This can only be achieved if participants know in advance which stimulus will appear on the next trial, or if they are in a stage of highly advanced implicit learning of the sequence. The presence of premature responses already in the first part of the learning session (**Figure 3A**) points to explicit rather than implicit origin of extremely speeded reactions. Hence, individuals from this group have discovered the presence of regularity during practice (online solvers) and have changed their mode of task processing from implicit to explicit (Robertson, 2007, 2009). This is confirmed by the unique pattern in these participants characterized by a dramatically speeded accumulation of sequence knowledge along with improvement of accuracy.

Another intriguing behavioral characteristic of this group was the increased performance variability. While increased variability in regular blocks can be explained with premature responses no such responses were generated in the first random block of learning, nor was RT specifically delayed in this group. Therefore, increased variability in the beginning of learning may not be directly related to checking of a perceived regularity (Frensch et al., 2002), nor may it index distractibility (Yordanova et al., 2011). Rather, this initial performance instability points to a different mode of processing in both random and regular blocks, independently of exposure to sequence. With regard to neurophysiological evidence for enhanced controlled processing during task encoding in subsequent solvers (Lang et al., 2006; Yordanova et al., 2009b; Darsaud et al., 2011) the unstable performance of online solvers identified here appears to reflect an active self-induced or self-instructed search for regularity. In line with previous reports (e.g., Nissen and Bullemer, 1987; Wagner et al., 2004; Haider et al., 2005; Yordanova et al., 2008) these results demonstrate that part of the individuals possess an inherent attitude to actively explore environmental structure.

#### Knowledge Awareness and Practice-Based Sequence Learning

In 8.2% of participants, a cognitive strategy was identified which promoted awareness on the basis of accumulated practice-based sequence knowledge. This strategy is represented by the SsK+/ExK+ sub-group. No signs of explicit sequence comprehension during learning (premature responses) were detected in this sub-group. On the background of progressive sequence learning, these participants presented with a unique performance feature, i.e., an enhanced performance variance only in the regular blocks, not in the random blocks, which occurred already with initial exposure to regularity in the first learning session. Also, enhanced variance to regularity was not synchronized with implicit gain progression in the course of learning. On these grounds, it may be suggested that in this sub-group, unstable performance in regular blocks reflects a strong penetration of an implicit model in a fragile form. On the other hand, it has been shown that during SRTT training, subjects do not learn uniformly all parts of a sequence (Schlaghecken et al., 2000; Wilkinson and Shanks, 2004). During exposure to regularity, parts of sequence can be consciously detected, while other parts remain a mixture of implicitly learned and unlearned fragments of the sequence (Miyawaki et al., 2005). It can be therefore also proposed that in this sub-group, increased variance to regular items results from partial explicit knowledge. This suggestion is consistent with previous observations (Yordanova et al., 2009a), according to which subsequent solvers in the NRT were characterized by a significantly larger RT variance of responses to predictable items, corresponding to a stronger activation of cognitive control brain regions (Darsaud et al., 2011).

#### Knowledge Awareness Promoted by Oﬄine Consolidation of Procedural Knowledge

A third sub-group of explicit solvers identified in the present study (12.8%) comprised participants who did not manifest enhanced performance variance and did not learn the sequence (SsK-/ExK+), thus showing a cognitive mode of processing very similar to that of explicit non-solvers who did not learn the sequence by practice (SsK-/ExK-). Major precursors of explicit knowledge generation in this sub-group, however, emerged after retention, when a substantial gain in sequence knowledge occurred along with increased performance variance. Obviously, oﬄine retention was critical for knowledge awareness in this sub-group (Wagner et al., 2004; Yordanova et al., 2008, 2009a,b, 2010). As indexed by high accuracy and exclusively stable performance at learning, these participants seem to have developed a fundamental focus on the overt level of SRTT, with overwhelming processing of stimulus-response (S-R) associations. This assumption is supported by another study of the same data set (Verleger et al., 2015). Increased parietal P3 components were yielded during SRTT learning in such participants reflecting intensive learning of S-R and R-S relationships, or additional testing of the feedback value of each stimulus (Verleger et al., 2015). Thus, current results suggest that delayed awareness of sequence can emerge on the base of firmly learned S-R pairs, i.e., consolidated overt SRTT level (Robertson, 2009; Yordanova et al., 2009a; Diekelmann and Born, 2010), and that the implicit penetration of regularity can act on consolidated S-R pairs. It remains to be established why oﬄine consolidation was efficient in promoting a subsequent integration of pairs in higher-order structures only in individuals with enhanced cognitive processing indexed by large P3 components (Verleger et al., 2015).

## Conclusion

(1) In implicit learning conditions, the formation of practice-based sequence representations precedes subsequent awareness of regularity. (2) Implicit sequence-specific knowledge alone is not a precursor of explicit knowledge generation. (3) A behavioral precursor of subsequent awareness is the combination between practice-based sequence knowledge and increased performance variance, pointing to an interaction between implicit and explicit processing systems during task practice. (4) Implicit-explicit interactions during task practice may have different origins: (a) inherent individual attitude to active exploration of environmental structure, (b) comprehension of fragmented sequence, or (c) interfering implicit representations. All cognitive modes contributing to awareness are marked by increased performance variability. These results may (i) refine the evaluation of online and oﬄine learning of tasks with dual structure, in particular SRTT, and (ii) extend our understanding of increased behavioral variability in both normal and pathological conditions.

## Conflict of Interest Statement

The authors declare that the research was conducted in the absence of any commercial or financial relationships that could be construed as a potential conflict of interest.

## Footnotes

1 The term “solvers” is used in the present study to designate individuals who become aware of the hidden task regularity. In tasks, such as the number reduction task (NRT, Haider and Rose, 2007), conscious awareness of regularity typically takes the form of insight (a sudden awareness of the hidden rule). Although the conscious discovery of regularity is not abrupt in the SRTT, we adopt the same designation for explicit knowledge generation in the SRTT.
